# Defining and measuring quality in acute paediatric trauma stabilisation: a phenomenographic study

**DOI:** 10.1186/s41077-019-0091-z

**Published:** 2019-04-11

**Authors:** Ralph James MacKinnon, Karin Pukk-Härenstam, Ulrica Von Thiele Schwarz, Christopher Kennedy, Terese Stenfors

**Affiliations:** 1 Department of Learning, Informatics, Management and Ethics, Karolinska Institute, Tomtebodavägen 18a, SE-171 65 Solna, Sweden; 2 Department of Paediatric Anaesthesia, Royal Manchester Children’s Hospital, Manchester, UK; 3 Paediatric Emergency Department, Karolinska University Hospital, Solna, Sweden; 4 School of Health, Care and Social Welfare, Mälardalen University, Västerås, Sweden; 5 Paediatric Emergency Department, Children’s Mercy Hospital Kansas City, Kansas City, USA

**Keywords:** Quality, Resuscitation, Trauma, Training, Emergency medicine, Paediatrics, Phenomenography

## Abstract

**Objectives:**

Trauma is the leading cause of death in children. The lack of an accepted definition of what constitutes a high-quality stabilisation of a traumatically injured child has limited the evaluation of direct interventions in simulation-based education and service-delivery models to improve trauma care. The aim of this study was to create a framework that delineates quality by exploring the perceptions of the multi-disciplinary team providing and improving this initial care.

**Methods:**

Interviews were conducted with 36 experienced UK trauma team members and governance administrators (clinical directors to executive board level), from three standard UK trauma units. This study used a phenomenographic approach to explore the relationships and hierarchy between the contrasting perceptions of quality and evaluation of quality in this acute context.

**Results:**

The findings show that defining quality is a more complex concept than simple proxy measurements, such as time to CT scanning. They also show that the concept of quality requires the consideration of a spectrum of perspectives that range from the simple to the more sophisticated.

This study highlights the importance of teamwork, individualised perspectives and the culture of care provision, when describing quality. A novel framework to delineate quality is presented, comprising System, Team, Process, Individual, Data and Culture.

**Conclusions:**

This study has created a framework of understanding of acute paediatric trauma care quality and its measurement from the perspectives of team members and administrators. A framework and future tools to capture and disseminate the System, Team, Process, Individual, Data and Culture perspectives of the quality of trauma stabilisations could be a key advance in the care of severely injured children.

**Electronic supplementary material:**

The online version of this article (10.1186/s41077-019-0091-z) contains supplementary material, which is available to authorized users.

## Introduction

Trauma is the leading cause of death in children [1]. The initial stabilisation of a traumatically injured child encompasses the primary assessment, lifesaving interventions, re-evaluation and, when needed, transfer to a major trauma centre [2]. High-quality trauma care depends on the knowledge, skills, attitudes and behaviours of the individuals that comprise a trauma team, team member interaction and health system organisation [2]. Over the past two decades, much attention has focused on simulation-based team training and the development of both technical and non-technical skills to improve resuscitation quality [3]. In situ trauma simulations, training trauma teams in their place of work, are becoming a normal practice in trauma centres across the globe [4]. Conversely, very little attention has focused on understanding the parameters defining high-quality initial trauma stabilisation. The understanding of what quality means in this context may be dependent on perspective, for example the perspective of a trauma team member, a team leader, an administrator or an educator.

This study used phenomenography, a research approach that captures and contrasts different ways of seeing, experiencing and understanding [5–8]. Phenomenography explores variation in perspectives and the architecture of this variation. It then seeks to improve understanding of how these perspectives are interrelated [5–8]. This provides a deeper understanding of a concept by incorporating multiple perspectives to create a clearer description.

To understand the rationale for this novel approach, we present a brief overview of the current concepts of trauma quality. Well-delineated quality indicators in trauma care have been challenging to develop, even more so in paediatric trauma care [9, 10]. To date, there has been no strong evidence for any clinical factor to serve as a quality indicator [10]. The approach used most often to remedy this lack of quality metrics is to create a system of proxy measures or task completion steps, such as time to intubation, used in the USA by the American College of Surgeons [11]. The data elements reported are based on adult resuscitation and do not necessarily reflect the needs of children [10]. In England and Wales, the Trauma Audit and Research Network (TARN), the independent monitor of trauma care, promotes improvements in care through a national comparative clinical audit [12]. The data elements focus on consensus-derived endpoints of care and include the number of patients meeting evidence-based guidelines regarding timings of computerised tomography (CT) scans, or system indicators of the presence of the most senior clinicians to lead trauma teams [12]. This data is useful for benchmarking endpoints of care in a health system but does not provide information about how the team should be trained or prepared to reach the targets. To date, this proxy approach to measuring quality has not been correlated with improved patient outcome or performance of teams. This approach to quality also lacks the granularity needed to define the recommendations an individual clinical unit needs to support high-quality trauma care.

At present, it is not clear whose understanding of quality is currently being relied upon and how the meaning of quality may differ between different stakeholders. As a result, there is no current articulation of what quality is, or how to evaluate it, and it is not known how quality relates to patient outcomes [13]. Without an understanding of quality in this context, and the ability to describe quality across team members and administrators, it is difficult to develop interventions to improve it.

The aim of this study was to create a framework of understanding of acute paediatric trauma care quality and its measurement from the perspectives of team members and administrators. This understanding of quality can inform simulation-based training, by targeting interventions towards knowledge, skills, mental model sharing [14] and team interactions, to improve care provision.

## Methods

### Study design and outcome measures

We used a qualitative phenomenographic approach. The phenomenon explored was defining and measuring quality in the initial stabilisation of a severely injured child. The outcome measure for phenomenographic studies is termed the “outcome space”. The outcome space is a description of the phenomenon created by comparing and contrasting perspectives, grouping these into categories and understanding how the categories are interrelated, in terms of any hierarchical relationship. The outcome space represents a description of the phenomenon based upon the perspectives of all the study participants.

A secondary numerical analysis of the outcome space was performed to further explore the different perspectives of the trauma team members and administrators.

### Study setting and recruitment

Thirty-six participants were selected from three hospitals in the United Kingdom (UK). The hospitals were standard UK trauma units, in that they did not provide definitive care for children. For severely injured children, the UK trauma units provide initial acute stabilisation prior to transfer to a major paediatric trauma centre. Six trauma team members and six administrators involved in trauma governance were enrolled in the study at each hospital (Additional file 1). The Emergency Department Clinical Director and the Medical Director of each hospital were directly contacted by telephone and then emailed by the first author (RM), and they provided contact details for trauma members of differing roles and administrators at the departmental, divisional and executive board levels of the organisations. All participants provided informed consent.

### Data collection

The study followed the consolidated criteria for reporting qualitative studies (Additional file 2) [15]. Data collection was by face-to-face interviews performed by the first author using a semi-structured interview guide (Additional file 3). The interviews lasted approximately 60 min and were recorded and transcribed verbatim.

### Phenomenographic analysis

A standard phenomenographic approach (Table 1) was used, with two modifications. Firstly, throughout the process, the comments of each participant were tracked and remained linked to the participants. Secondly, a numerical analysis of the perspectives was undertaken. The rationale for the modifications was to explore in more detail the differences in perspectives between clinicians and administrators. All of the transcripts were read thoroughly by two researchers (RM & TS). One researcher (RM) read each transcript again and marked the sentences in which the participant described what quality of a paediatric trauma stabilisation meant to them and how they suggested this quality could be measured (familiarisation stage). Descriptive “meaning units” of one to three words were taken from each sentence (condensation stage). The meaning units were then compared for similarities and contrasted for differences (comparison stage). The meaning units that expressed similar ways of understanding the phenomenon were allocated to the same category (grouping stage). The perspective captured by each category was described (articulation stage). The essential meaning of each category was explored by checking the meaning units by re-reading each whole transcript, to ensure each participant’s perceptions were correctly identified (labelling stage). Each category was challenged to ensure that it was qualitatively distinctive and had the minimum number of meaning units that could capture all the variations of perspectives within that category [16–18]. After this, the first author returned to the data and repeated the above process until a consensus was reached with the research team (contrasting stage). This iterative process resulted in some categories being dropped in the final analysis. The final step in establishing an outcome space that defined the phenomenon was to investigate the internal relations between the categories and explore any hierarchy of understanding.

**Table 1:** The seven stages of phenomenographic analysis [7]

1. Familiarisation	Reading through all interview transcripts in depth to get an impression of how the interview proceeded. All data (viewpoints) in the entire pool are given equal consideration.
2. Condensation	Identifying meaning units in the dialogue of each interview and marking or saving these for further scrutiny.
3. Comparison	Comparing each of the meaning units for similarities and differences.
4. Grouping	Allocating answers expressing similar ways of understanding the phenomenon to the same category.
5. Articulating	Capturing the essential meaning of a certain category.
6. Labelling	Expressing the core meaning of each of the categories. Steps 3–6 are repeated in an iterative procedure to make sure that the similarities within and differences between categories are established.
7. Contrasting	Comparing the categories through a contrastive procedure whereby they are described in terms of their individual meanings as well as in terms of what they do not comprise.

Throughout the above process, the meaning units within the categories remained linked to the participants. This allowed the meaning units expressed by each participant to be mapped to the final categories. A map of the meaning units of each participant was created. The maps were directly visualised and organised according to the number of categories expressed by the participants. A hierarchy of increasing complexity, composed of four levels, was identified. Level 1 comprised participants that included perspectives from three categories when describing the phenomenon. Level 2 included perspectives from four categories. Level 3 included perceptions from five categories. Level 4 included all of the six categories described by the study participants. An audit trail of the analysis is presented in Additional file 4.

### Numerical analysis

All of the meaning units within the categories were linked to participants. The categories referred to by each participant, clinical members of the trauma team and administrators, were counted.

### Reflexivity

Reflexivity is a state of continual awareness and understanding on the part of research team members that their prior experiences and/or assumptions may influence all aspects of the study [16]. A number of steps were taken to foster a reflexive research study design (Additional file 5). The steps included a continued reflexive dialogue between the five international researchers with differing backgrounds and understandings of the study phenomenon, a visible audit trail of data that promoted reflection on reflexivity and openly exploring reflexivity of the preliminary data, at an international research meeting.

## Results

The results of a phenomenographic study are presented as structural categories of perspectives and a hierarchy of the variation of perspectives. Each structural category is presented with sub-categories, termed referential categories, derived from the individual meaning units from all participants (Table 2). The hierarchy described is from a basic to a more comprehensive understanding of quality and how it can be measured. At each level of the hierarchy, more categories are incorporated. At the most basic level, only three structural categories are present; at the most complex, there are six.

**Table 2:** The perspectives of the quality and measurement of acute stabilisation of a traumatically injured child

Structural categories of perspective of quality	Referential categories of perspective of quality
System: the organisational design to facilitate optimal performance.	Ready/pre-planned, critical incident reporting systems, equity of care, standards, prioritisation, value for money, feedback to team, feedback from major trauma centres, coffee room feedback, current lack of tools to measure quality, team-working tools, friends and family test during stabilisation, checklists, cognitive aids, audit.
Team: the mechanics of how the team functions.	Teamwork, leadership, communication, team satisfaction, supported teams, team performance monitoring, ongoing team training.
Process: the direct delivery of care to the patient.	Best care provision with resources available, best evidenced, following protocols (Advanced Trauma Life Support, European Trauma Course), timelines.
Individual: the innate personal perspective of healthcare providers.	Internal assessment by team members, personal desire, personal satisfaction, specifically trained/experienced, patient’s experience, patient-centred, safety of patient, perception of carers/parents.
Data: the facts and details collectable for analysis.	Patient outcomes (morbidity, mortality), adverse clinical events (sudden untoward incidents), clinical data, Trauma Audit Research Network data (timings), electronic patient record, retrospective note reviews, benchmarking against other hospitals.
Culture: the social behaviour and customs of the team and organisation.	Debriefings post-resuscitation, reflective practice, guardians/champions of quality, inter-professional discourse, approachability of senior clinicians.

Direct quotations from the interviews are provided as examples and presented in italics. Also presented are the most frequent perspectives of clinicians and administrators (Table 3).

**Table 3:** The most frequent perspectives of trauma team members and non-clinical trauma governance administrators defining quality of care and measurement

Trauma team members	Non-clinical administrators	Structural category
Teamwork	Team performance monitoring	Team
Current lack of tools to measure quality		System
	Patient outcomes	Data
Best care process with resources available		Process
	Debriefing	Culture
Internal assessment	Safety of patient	Individual
Friends and family test during resuscitation		System
Process timelines		Process

### Perspectives of quality and ways of measuring quality

Six structural categories were identified to describe the participants’ varying perspectives of quality and ways of measuring quality. The categories are System, Team, Process, Individual, Data and Culture (Table 2).

#### The System perspective

This perspective of quality focused upon how ready the hospital was to receive traumatically injured children, in terms of standards of care, infrastructure and the ability to provide feedback on the care provision to optimise future performance.

So, quality to me means that there is, there has been pre-planned organisation of how to manage a paediatric trauma patient, so that there are protocols in place at the hospital, so there is a system in place. (Consultant General Surgeon).

The current absence of tools to evaluate quality was referred to by many participants.

There is no (quality) measurement tool. I think it is just sort of word of mouth between people that have been put in a certain scenario of saying, you know we did not have this, or that did not go well, or we needed this...then feeding it back to the consultants and try to get some changes made. (Emergency Department Paediatric Nurse).

Tools that could evaluate quality that was discussed were critical incident feedback systems, audits, checklists, team-working tools and a friends and family test. The friends and family test asks people to consider whether they would recommend the care being provided at that moment to a friend or family member [19]. However, in the absence of other sources of formalised or structured feedback on performance, many participants reported coffee room feedback or using the friends and family test during a stabilisation, as a quality measure.

I have been in situations where I have thought that I am glad it is not my child in front of me being managed like this...yes, we could use that as a measure of quality. (Emergency Department Consultant).

#### The Team perspective

The Team perspective was described in a number of ways. These included the well-understood concept of teamwork, with leadership and communication as key features of a high-quality trauma stabilisation. However, team performance monitoring as a way to determine quality and the inherent difficulties in evaluating teamwork were also emphasised.

I suppose (quality) is ensuring that the team works as a unit together to ensure the best outcome for that patient. So, everybody works together, there is a leader that takes charge and directs the team, somebody makes decisions at the appropriate time. (Operating Department Practitioner).

Team performance is a hard quality thing to measure. There is not anything to capture or review (team performance) unless somebody within the team puts in an adverse incident form to say, I do not know, the Trauma Team Leader was rubbish or none of the laryngoscope blades were working because the batteries were dead or there was no carbon dioxide monitoring so we put the tube down the oesophagus and we did not realise what we had done. Unless something like that comes out of it, you would only know that a traumatically injured child came in and had a good outcome. You would not know it was a war within the team as it was going on. (Emergency Department Consultant).

#### The Process perspective

The process was described as how the injured child was treated, both in terms of adherence to well-established protocolled trauma care, such as Advanced Trauma Life Support or European Trauma Course guidelines/protocols, and the time taken to provide this care.

So I think the quality of a good resuscitation would be indicated by a stable patient getting treatment as quickly as possible...I think you measure it (quality) in that you have achieved stable vital signs, you have made the patient safe, so you have followed the ABC approach, that investigations are done in a timely manner and the patient is in the trauma centre as quickly and as safely as you can do that. (Consultant Anaesthetist).

This quality perspective included the TARN data points, currently used as measures of quality including times to senior review, CT scanning and intubation. The majority of participants understood quality as more than hitting target times, but reported that, as these times can be easily measured, this is what is used.

Trauma audit research network times to senior, time to CT, time to transfer, they are good measures of quality…(but) there are a lot of external factors that influence these. (Paediatric Emergency Department Consultant).

There’s easy things to measure, I guess, like time to CT scan, time to intubation, the time to you know, but within those, there are lots of complex steps, such as decision to intubate, the decision to order a CT scan or not, or do the fast scan before the CT scan, and capturing that level of data is poor, I think because it depends on clinicians inputting that data in their notes and that we know as clinicians is pretty ropey, particularly in these serious cases where things tend to go ahead of the note-taking, so no I am not sure you necessarily have the equipment necessary to capture the data required to make that level of assessment of providing quality. (Divisional Level Head of Trauma).

You have a very complex team that are working together...the easiest thing to measure is the process time. (Intensive Care Consultant).

#### The Individual perspective

The Individual perspective captured the concept that individuals providing the care during the acute stabilisation and immediately afterwards consider quality and evaluate it on a personal level. Individuals emphasised that their own direct assessment of care, as measured against their own personal standards, was an important indicator of quality. Participants indicated that the care they personally provided during the stabilisation, whether optimal or not, directly determined how much personal satisfaction they drew from the experience.

Quality, I guess personally what we would be striving to do is to give the seriously injured child the care I would hope for my own children, should they arrive on a trauma centre doorstep…I think as clinicians, you can walk into disasters and you just think oh my God, this is awful, we need to do something about this…the feeling is a very personal thing and I do not know how you would measure it. (Trauma Lead, Emergency Department).

It (quality) is a personal feeling, I think, as to whether you felt it ran well. I mean I can look back over my time, I can think of times when you think of a great trauma stabilisation, and it went really well and it was really good, the team worked well, and I can think of ones that you think actually that did not go well, but that’s personal things and you kind of congratulate yourself and the team on the good ones…but that’s a feeling rather than anything else, it seems to me. (Surgical Specialist Trainee).

It’s anecdotal but I think as a clinician it’s a feeling you have, and the nurses do and everyone in the department...the initial feeling after something has happened, did that go well or yes that should have gone much better. (Paediatric Specialist Trainee).

Well I would not say that we measure quality at all, we I think as individuals formally assess it. I will analyse my own performance in the car on the way home. (Divisional Level Trauma Lead)

#### The Data perspective

The Data perspective captured the viewpoint that quality is data-driven, focusing on patient outcome (mortality or morbidity), adverse events (sudden untoward or critical incidents) and currently recorded data.

(Quality) means a good outcome at the end of the day...of course you can be more specific and say time through doors, time of first primary assessment…to any particular sort of scanning or intervention, to transfer time...you can use time as a marker, but it’s not the whole story...yet these are things that we can measure. How relevant they are only can be seen in time, when we have pooled data for what is a relatively rare event. (Divisional Level Clinical Director)

This perspective also included benchmarking to compare the provision of quality with other hospitals and the need for adverse incident data.

Most hospitals tend to look at outcomes rather than processes; if you got a good outcome it will not trigger a sudden untoward critical incident on morbidity or mortality, unless somebody puts in an untoward incident because the management was so shocking. (Anaesthesia and Intensive Care Consultant)

#### The Culture perspective

The final perspective of quality focused on culture. This perspective highlighted debriefing, learning through reflective behaviour and the presence of champions, within the team promoting this, as an integral part of quality. The evaluation of this was suggested as a potential quality metric.

We clearly need to get smarter and more reflective…in other high-risk industries there is much more freedom and space to talk about teamwork. Fundamentally it’s just part of your job, you get trained in giving facilitated feedback, you have to do it at the start and finish of each shift, it is non-negotiable, you can raise issues and concerns without fear of retribution. There is still a culture gap to bridge where the airline and nuclear industry are in terms of culture change. I mean there have been big steps in the NHS in the last ten years, but we are still nowhere near there. (Divisional Level Director)

This perspective highlighted the fact that quality includes the presence of guardians or champions that promote reflection and inter-professional discourse.

There has to be a kind of innate reflection by individuals and leadership. By individuals to say hang on we have got a good outcome despite us making a real mess of things, that is because it would not be captured, so you have to rely on the professionalism of the individuals that messed up (to declare this) and by the grace of God have had a good outcome. (Executive Board Level Medical Director).

Finally, this perspective highlighted the fact that debriefing post-trauma resuscitation provides the potential opportunity to define and enhance quality but is currently difficult to organise.

You could do a survey at the end of every kind of situation, if you did a debrief. You could kind of discuss quality and maybe everybody has an anonymous questionnaire to fill in…I have not been involved in any debrief and I have never been involved in any kind of questionnaires regarding what happened in these situations, so from my perspective I am not aware of it happening. (Operating Department Personnel)

### Hierarchy of perspectives

As stated earlier, the hierarchy described is from a basic to a more comprehensive understanding of quality. At each level of the hierarchy, more categories are incorporated. The simplest understanding of quality and ways of measuring it was provided by participants who utilised units of meaning from three structural categories: System, Team and Process. Participants with a more comprehensive understanding included an additional category, the Individual. The fifth category included by participants was Data. The most complex understanding was provided by participants who described quality and ways of measuring it by adding units of meanings from the Culture category.

### Perspectives of clinicians and administrators

The perspectives of clinicians and administrators are presented by tabulating the frequency of categories referred to in their description of quality and measurement (Table 3). Comparing these perspectives, there was a shared view on the importance of teamwork and team performance monitoring, and a concurrent lack of tools to measure quality and provision of the best care process with the resources available. The administrators’ views on the importance of patient outcomes, debriefing and safety were not shared by the team members. The team members focused more upon internal assessment, process timelines and use of the friends and family test (during stabilisations) as defining and measuring quality.

## Discussion

### Interpretation of the main findings

The aim of this study was to create a framework of understanding of acute paediatric trauma care quality and its measurement from the perspectives of team members and administrators. This study has highlighted that defining quality is a more complex concept than simple proxy measurements, such as time to CT scanning. The concept of quality requires a consideration of a spectrum of perspectives. The spectrum ranges from simple to more sophisticated understandings. The ways of understanding quality were independent of being a clinician or an administrator. This understanding of the complexity of quality has implications for future simulation-based training, debriefing and performance reports of hospital readiness to receive patients. Such an understanding of quality could also greatly enhance quality assurance and improvement discussions among clinical teams, educators and administrative staff.

The findings of this study highlight six potential aspects of quality of the acute stabilisation of a traumatically injured child, as well as highlighting the current lack of methods for measuring quality. The six perspectives of quality reported are System, Team, Process, Individual, Data and Culture.

#### System engineering and human factor principles

The basic level of quality defined in this study by Process, Team and System shares elements of established healthcare quality models. The classical Donabedian Structure, Process and Outcome (SPO) model describes Structure as “organizational factors affecting the context in which care is delivered”, Process as the “care delivery factors” and Outcome as including “all effects of healthcare on patients” [20, 21]. The Systems Engineering Initiative for Patient Safety (SEIPS) model builds on the Donabedian model but focuses on describing the interconnectedness of the system and interactions that impact patient safety and employee/organisational outcomes [22]. A previous scoping review of indicators in trauma care failed to find any strong evidence for a single clinical factor as a quality measure and supported an SPO model approach to understanding quality in this setting [9]. The multi-faceted approach of these models is aligned with the perceptions of the participants in this study. This study also supports the importance of placing individuals at the centre of a work-based system aiming to enhance performance. However, where the SEIPS model has quality as an undefined outcome measure, this study sheds light on what constitutes quality in this acute setting by delineating a framework of quality with six potential quality markers.

The findings of this study are also aligned to the current understanding of the importance of a “human factors” approach to improving trauma care [23–25]. In this study, the Team category is at the simplest level of the hierarchy, along with System and Process. This is in contrast to the System, Process and Outcome approach of the SPO and SEIPS models. The findings from this study indicate the need to consider additional perspectives, namely Individual, Data and Culture. Perhaps to truly understand and measure quality, it is necessary to consider not only what is done to achieve an outcome but also how the outcome is achieved.

#### Implications for the future measurement of quality

Many participants in this study emphasised that quality is not measured because there is a lack of tools to do so. The design of a tool to measure quality in this context may be aspirational at present. A number of questions need to be answered before this can be developed. Firstly, is it possible to measure quality at all, during the stabilisation of an acutely injured child? Secondly, are we missing other important perspectives, namely those of patients and families? And finally, can we design a tool that speaks to the needs of trauma team members, educators and administrators, all of whom are aiming to enhance the quality of care provided? These are important questions prior to considering the detailed design and subsequent validation of a simulation or workplace-based assessment.

The findings from this study would indicate that measurement of quality is possible.

Somewhat unexpectedly, many clinicians stated that they used the friends and family test to determine the quality of care provision during stabilisations. This is a very simple tool, often a traffic light system, to indicate that care was good, bad or acceptable. This tool is usually reserved for patients or families on leaving hospital. To our knowledge, the use of the friends and family test by health professionals to measure care in an acute setting has not been previously reported. The use of this test may be reflective of the lack of any other tool; however, it does indicate that it is possible to assess quality with a simple tool during the provision of care in this setting. It is reasonable to surmise then that a more comprehensive tool could be developed.

The use of a tool normally reserved for families brings us to consider the potential patient or consumer perspective in designing an optimal quality tool in this context. When evaluating the quality of nursing care, patients have previously emphasised the importance of technical and task-orientated skills in addition to respectfulness and caring [26]. A central precondition for quality of care, as judged by patients, was training and adequate resource provision [26]. The value of in situ simulation-based trauma training and how this contributes to the quality of care provided by a hospital, from the perspective of patients, remains unclear. At present, we can only speculate as to the extent that patients know about how hospitals train staff and their views on this. Undoubtedly, the perspective of patients is a key future step when developing staff training programmes and service quality tools.

To determine whether it is possible to design a tool that speaks to the needs of trauma team members, educators and administrators, there are a number of considerations. One such concern is the importance of communicating quality across stakeholders [27]. The six quality perspectives reported in this study provide a valuable step to a common language and goals for clinicians and administrators. There are similarities but also differences in views. Both clinicians and administrators emphasised the importance of teamwork and monitoring as defining aspects of quality. In simulation-based research, there are a plethora of tools to study teamwork. However, to our knowledge, these are not routinely used in clinical practice. Contrasting perspectives were also evident: there was a more frequent focus on patient outcomes, debriefing and patient safety on the part of administrators, whereas clinicians were more likely to focus on internal assessment and process timelines. This in part may be due to a more practical clinical perspective from “the shop floor”. One example of the constraints highlighted by participants was limiting debriefings due to staff being called away or transporting the patient onwards. The emphasis on patient safety highlighted by administrators may have been inherent in the considerations of clinicians, but this was perhaps less emphasised due to a focus on trauma care at interview. Safe patient care is the cornerstone for high-quality care. We postulate that the findings of this study are a step towards a tool that captures both the negative and positive aspects of the work system [22]. With an emphasis on Team, Individual and Cultural perspectives of the work as carried out in Emergency Departments [13, 28], there is alignment with the current direction of safety research [28].

An important area for further study includes the development of a multi-faceted tool that positions individuals at the centre of the work-based system with the ability to assess and reflect on the quality of the acute trauma care they provide. The utilisation of such a tool to train teams and monitor clinical performance could bridge both simulation-based training in the workplace and clinical care delivery. The inclusion of a quality framework based on the six perspectives reported in this study, within such a tool, will provide both the goals to train teams towards and direction for future metric development.

### Study strengths and limitations

The phenomenographic study design has deliberately illuminated a wide range of different perspectives. The equity of the importance of each individual perspective has been maintained throughout. No particular individual or group of perspectives has been valued more highly than others. No perspectives have been taken as sole arbiters of the truth regarding what quality means. Equal focus has been applied to the minutiae of thoughts expressed at interview. This study has delineated quality in this context, in a framework that speaks to all stakeholders.

One limitation of the study is transferability of the findings globally. All participants in this study work in hospitals in the same region in the UK. However, the findings have been presented to both researchers and clinicians in the field nationally and internationally, to enhance transferability.

A second limitation is the absence of patient perspectives in delineating the quality and measurement of a paediatric trauma stabilisation. Public and patient engagement was sought at the initial design stage of this study. Previous patients were selected by the research site Patient Public Involvement team in conjunction with the Trauma Rehabilitation nursing team. Of the six families contacted, none were able to attend the planned evening event. Work is currently underway to engage optimally with trauma patients and families, and this remains a direction of travel for the future design of a quality measurement tool.

## Conclusions

This study has created a framework of understanding of acute paediatric trauma care quality and its measurement from the perspectives of team members and administrators. It has highlighted that defining quality is a more complex concept than simple proxy measurements, such as time to CT scanning. It has also highlighted that the concept of quality requires the consideration of a spectrum of perspectives that range from simple to more sophisticated ways of understanding. The importance of teamwork, individualised perspectives and the culture of care provision, when delineating quality, has been emphasised by both trauma team members and administrators, in addition to System, Process and Data. An understanding of the complexity of quality is the key to future simulation-based training, debriefing and performance reports of hospital readiness to receive patients. The capability to capture and disseminate the System, Team, Process, Individual, Data and Culture perspectives of the quality of trauma stabilisations could be a key advance in the care of severely injured children.

## Additional files

Additional file 1: The study participants (DOCX 13 kb)

Additional file 2: The consolidated criteria for reporting qualitative studies (COREQ): 32-item checklist (DOCX 23 kb)

Additional file 3: The semi-structured interview guide (DOCX 25 kb)

Additional file 4: The phenomenography data audit trail (DOCX 1553 kb)

Additional file 5: Paediatric trauma educational interventions targeted to directly improve patient care—participant information sheet (DOCX 82 kb)

## Abbreviations

CD: Clinical director
CT: Computerised tomography
ED: Emergency department
MD: Medical director
NHS: National Health Service
SEIPS: Systems Engineering Initiative for Patient Safety
SPO: System, Process, Outcome
TARN: Trauma Audit and Research Network
TU: Trauma unit
UK: United Kingdom
