# Supplementary material for: Defining and measuring quality in acute paediatric trauma stabilisation: a phenomenographic study
Source: Adv Simul (Lond). 2019 Apr 11;4:4. doi: 10.1186/s41077-019-0091-z (PMC6458622; doi:10.1186/s41077-019-0091-z)
Supplement: Supplementary file 1 — The study participants (DOCX 13 kb) [file 41077_2019_91_MOESM1_ESM.docx]

# Additional File 1. The Study Participants

| **Trauma Team Members** | **Trauma Quality Administrator/ Manager** |
| --- | --- |
| Specialist Trainee Anaesthesia (Resident Anesthesiologist) | **Hospital Organisational Tier 1 Executive Board Level** |
| Senior Emergency Department Nurse | Medical Director |
| Emergency Department Nurse | Medical Director |
| Operating Department Personnel (Assistant to Anaesthetist) | Director of Nursing & Quality |
| Emergency Department Paediatric Nurse | Director of Nursing |
| Consultant Anaesthetist (Anesthesiology Attending) | Deputy Medical Director |
| Specialist Trainee General Surgery (Resident General Surgery) | Director of Performance |
| Emergency Department Consultant (Emergency Department Attending) | **Organisational Tier 2 Divisional Board Level** |
| Specialist Trainee Paediatrics (Resident Paediatrics) | Divisional Head of Trauma |
| Operating Department Nurse (Assistant to Anaesthetist) | Divisional Trauma Lead |
| Specialist Trainee General Surgery (Resident General Surgery) | Divisional Director of Surgery |
| Specialist Trainee Emergency Medicine (Resident Emergency Medicine) | Clinical Director of Emergency Medicine |
| Emergency Department Paediatric Nurse | Clinical Director of Anaesthesia |
| Specialist Trainee Emergency Medicine (Resident Emergency Medicine) | Trauma Lead & Clinical Director Emergency Medicine |
| Specialist Trainee Anaesthesia (Resident Anesthesiologist) | **Tier 3**  **Departmental Level** |
| Senior Emergency Department Nurse | Trauma Lead (Surgery) |
| Consultant Intensivist (Attending Intensive Care Medicine) | Paediatric Lead (Emergency Medicine) |
| Emergency Department Consultant (Emergency Department Attending) | Trauma Lead (Paediatrics) |
|  | Trauma Lead (Anaesthetics) |
|  | Trauma Lead (Emergency Medicine) |
|  | Trauma Lead (Emergency Medicine) |
|  | Paediatric Trauma Lead (Emergency Medicine) |
