# Supplementary material for: Defining and measuring quality in acute paediatric trauma stabilisation: a phenomenographic study
Source: Adv Simul (Lond). 2019 Apr 11;4:4. doi: 10.1186/s41077-019-0091-z (PMC6458622; doi:10.1186/s41077-019-0091-z)
Supplement: Supplementary file 2 — The consolidated criteria for reporting qualitative studies (COREQ): 32-item checklist (DOCX 23 kb) [file 41077_2019_91_MOESM2_ESM.docx]

# Additional File 2. The consolidated criteria for reporting qualitative studies (COREQ): 32-item checklist

(https://academic.oup.com/intqhc/article/19/6/349/1791966/Consolidated-criteria-for-reporting-qualitative) last accessed 06 July 2017

| No | Item | Guide questions/description |
| --- | --- | --- |
| Domain 1: Research team and reflexivity |  |  |
| Personal Characteristics |  |  |
| 1. | Interviewer/facilitator | First author |
| 2. | Credentials | BSc (hons),MB CHB, FRCA |
| 3. | Occupation | Paediatric Anaesthetist & Intensive Care Transport Physician |
| 4. | Gender | Male |
| 5. | Experience and training | Interviewer was trained and coached by last name author and practiced interview skills at a base hospital with the interview guide. |
| Relationship with participants |  |  |
| 6. | Relationship established | The interviewer does not work as staff at any of study sites, but the interviewer may have met individuals during clinical transport work (although does not recall this) or during medical training for 30 years previously. |
| 7. | Participant knowledge of the interviewer | The interviewer was a member of the regional transport team that served each hospital and the team would have been known to some of the participants. The transport team retrieves patients from thirty hospitals, in the region in which this study was set. There were seven consultants on the team, at the time of the study. |
| 8. | Interviewer characteristics | The interviewer has a strong interest in paediatric trauma simulation-based education and was self-aware of biases and assumptions that could influence this research. |
| Domain 2: study design |  |  |
| Theoretical framework |  |  |
| 9. | Methodological orientation and Theory | The methodological orientation to underpin the study was phenomenography. |
| Participant selection |  |  |
| 10. | Sampling | The participants were purposively selected for a wide variation of perspectives. |
| 11. | Method of approach | The clinical directors (CD) and medical directors (MD) at each site were contacted by email then phone by the interviewer. Each CD & MD suggested hospital staff of diverse roles to approach. Participants were initially approached by phone call and then by email to provide the participant information sheet and consent forms. Informed consent was taken prior to the interviews taking place. |
| 12. | Sample size | Thirty-six participants |
| 13. | Non-participation | No refusals. |
| Setting |  |  |
| 14. | Setting of data collection | The data was collected at the participants place of work, with two exceptions, in each case the interview was conducted face to face in the participant’s home. The interviewer was invited to the participant’s home after it was considered not possible to meet at work because of time constraints. |
| 15. | Presence of non-participants | No one anyone else was present besides each participant and the researcher. |
| 16. | Description of sample | The sample comprised of thirty-six purposively sampled participants from three hospitals, each representative of all general hospitals in the region of study. Eighteen of the participants were from trauma teams (all team member roles, disciplines and seniority level) and eighteen were from trauma governance boards at three levels, departmental, divisional and executive board level. The role of each of the participants is indicated in Additional File 1. The interviews were conducted over a five-month period. |
| Data collection |  |  |
| 17. | Interview guide | An interview guide (Additional File 3) was created, tested with pilot interviews at the base hospital of the first author. The pilot recordings were transcribed and discussed by the research team. The interviewer then practised with the agreed guide. The data from the pilot study was not included in this research. |
| 18. | Repeat interviews | No repeat interviews were carried out. |
| 19. | Audio/visual recording | This study used audio recording to collect the data. |
| 20. | Field notes | Field notes were made during and after the interviews. |
| 21. | Duration | The duration of the interviews was approximately sixty minutes. |
| 22. | Data saturation | Data saturation was discussed; however, a decision was made to complete all interviews to capture as much variation as possible for phenomenographic analysis. |
| 23. | Transcripts returned | The transcripts were not returned to participants for comment and/or correction. |
| Domain 3: analysis and findings |  |  |
| Data analysis |  |  |
| 24. | Number of data coders | Two data coders coded the data, the first and last name authors. |
| 25. | Description of the coding tree | The authors have provided a description of the phenomenographic analysis from the steps of familiarisation to contrasting and have provided a step wise audit trail in photographic form (Additional File 4) Further anonymised data can be provided on request. |
| 26. | Derivation of themes | A framework was derived from the different participant perspectives explored by the phenomenographic methodology. |
| 27. | Software | No software was used to manage the data. |
| 28. | Participant checking | The participants did not provide feedback on the findings. |
| Reporting |  |  |
| 29. | Quotations presented | Participant quotations are presented to illustrate the findings. Each quotation is identified by the participant role. |
| 30. | Data and findings consistent | There is consistency between the data presented and the findings. |
| 31. | Clarity of major themes | The major themes, termed structural categories are clearly presented in the findings. These are subdivided into referential categories that are also presented. |
| 32. | Clarity of minor themes | All perspectives were analysed and clearly presented. |
