# Supplementary material for: Defining and measuring quality in acute paediatric trauma stabilisation: a phenomenographic study
Source: Adv Simul (Lond). 2019 Apr 11;4:4. doi: 10.1186/s41077-019-0091-z (PMC6458622; doi:10.1186/s41077-019-0091-z)
Supplement: Supplementary file 3 — The semi-structured interview guide (DOCX 25 kb) [file 41077_2019_91_MOESM3_ESM.docx]

# Additional File 3. The Semi-structured Interview Guide

# Introduction

Thank you for your time.

(italics is an aide memoire):

(*Ensure consent forms are signed and information given reminding participants that they can withdraw at any time and that they are being recorded*)

This interview is about quality improvement within your hospital as a trauma unit.

Are there any questions before we get started?

# Questions

## Quality Improvement in paediatric trauma

I would like to present two scenarios to you, one is a seven-year old hit by a car suffering severe injuries and is managed well by your trauma team and the child does well. The second is a seven-year old also hit by a car, same injuries but is not managed well and does not do well (severely disabled).

I would like to explore what happens (*or you feel should happen – if interviewee does not know*) after each of these cases:

Regarding case one and the team members present who managed the child - what would happen afterwards?

Regarding case 1 and those not present (clinical and non-clinical) what would happen?

Then, regarding case 2, where management was not good, and the outcome was poor:

Regarding the team members that were present – what would happen?

Regarding those not present (*clinical and non-clinical*) what would happen?

## The Quality Improvement (QI) review process

*Assuming that a review process is undertaken:*

Can you please tell me the purpose of the review process?
1. To report / register ?

2. To learn ?

(*formally what the process is about versus perceived about*)

Does this feed into your hospital QI process, if so how?

Can you help me to understand / map the review (*post trauma care*) process?

Who would be involved?

Can you describe the tools you use? (data used, verbal, documents – from whom)

Do you / capture /have all the data required to learn from experience?

When would this happen? Describe the setting / time from when child presents

Can you provide a real example of this occurring in your trauma bay, if not the trauma bay then somewhere else in your Emergency Department (ED).

We have discussed one way that you work with QI in your Trauma Unit (TU) – are there any other processes?

Is there a QI team? How are they related to ED staff (same people?) If not who?

Do you have QI activities integrated in everyday work?

Do you have time set aside for QI work?

Is there specific QI training for team & staff?

Are there QI goals set by the managers? – what are these – do you have an example?

How do you see your role in the improvement work in your TU?

What do you need to allow you to do this? – (in terms of support / processes/ information)

If you wanted to change anything about the way the hospital manages trauma care today, how would you go about it? What opportunities are there?

Are QI goals aligned with other performance goals?

Do you consider senior management view that Qi work is important? (*Do you? How?*)

You have explained that you have these mechanisms for learning, do you have any other ways have for reflection and learning about practice in the hospital today?

(What other ways have you for reflection and learning about practice in the hospital today).

## Looking from a broader perspective

What does quality mean to you, in terms of managing traumatically injured children?

How do you measure that quality at present?

What other measurements do you think may be relevant?

Are there aspects of quality that cannot be measured?

What are the barriers/challenges you are currently experiencing regarding paediatric trauma care at this unit?

Reflecting back on those 2 scenarios – what are your thoughts if I were to say the team that had the good outcome were completely dysfunctional and a superb team had the poor outcome?

Does this matter?

How would your hospital review this?

# Closure

Do you have any other feedback that you would like to share with me?

Thank you again for your time.
