# Supplementary material for: Defining and measuring quality in acute paediatric trauma stabilisation: a phenomenographic study
Source: Adv Simul (Lond). 2019 Apr 11;4:4. doi: 10.1186/s41077-019-0091-z (PMC6458622; doi:10.1186/s41077-019-0091-z)

# Additional File 4. The Phenomenography Data Audit Trail

The phenomenon explored was defining and measuring quality in the initial stabilisation of a severely injured child.

## Familiarisation, Condensation and Comparison Stages

These stages resulted in meaning units, each expressing perspectives of the phenomenon.


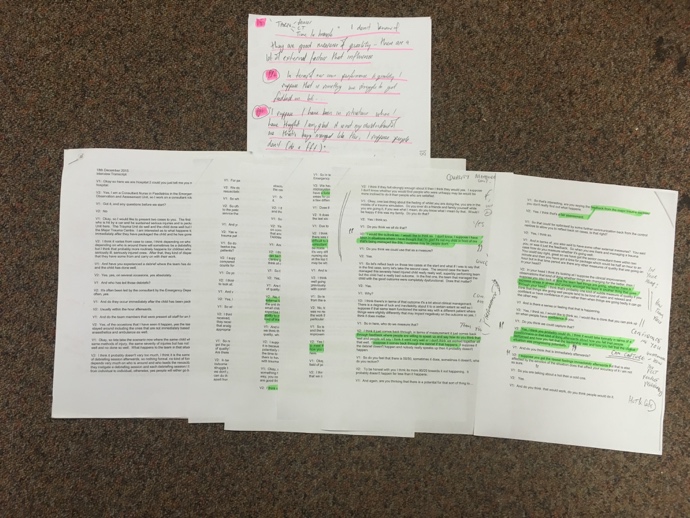


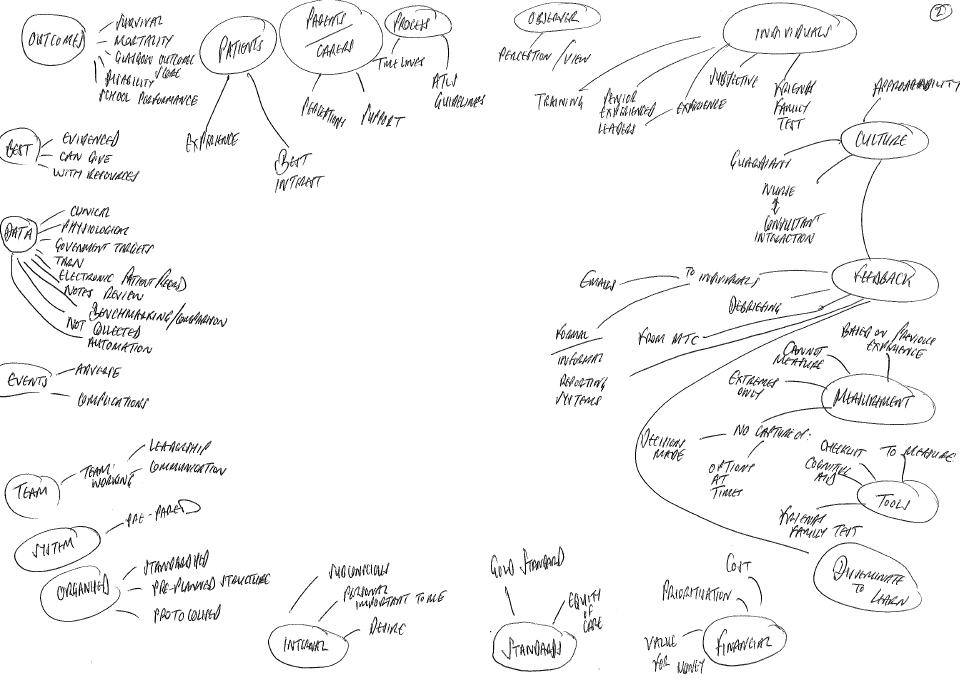


## Grouping, Articulation, Labelling and Contrasting Stages

This resulted in categories that were qualitatively distinctive, with a minimum number of structural linked categories that could capture the variations in experience or conceptions. Preliminary categories were dropped, the final pathway is shown.


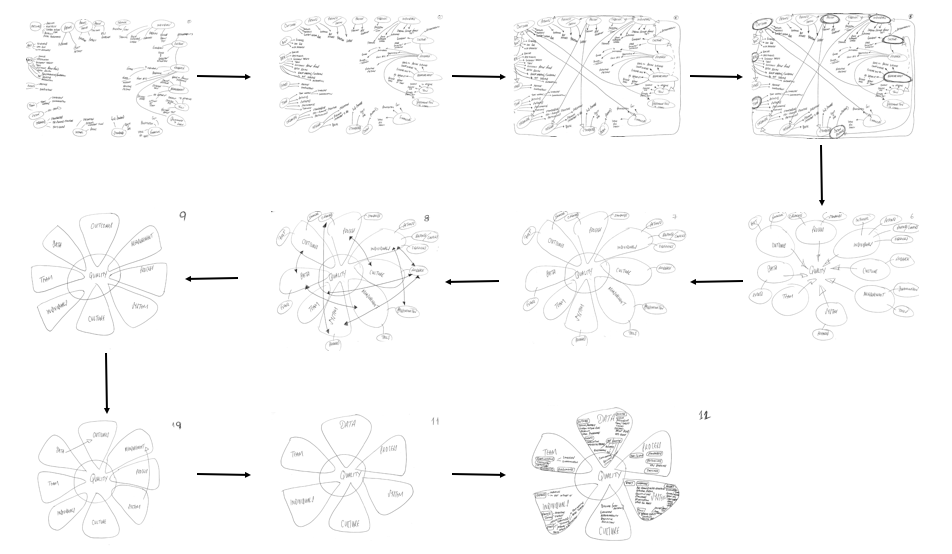


## Meaning units linked to perspectives

This resulted in meaning units trackable to participants throughout the study.


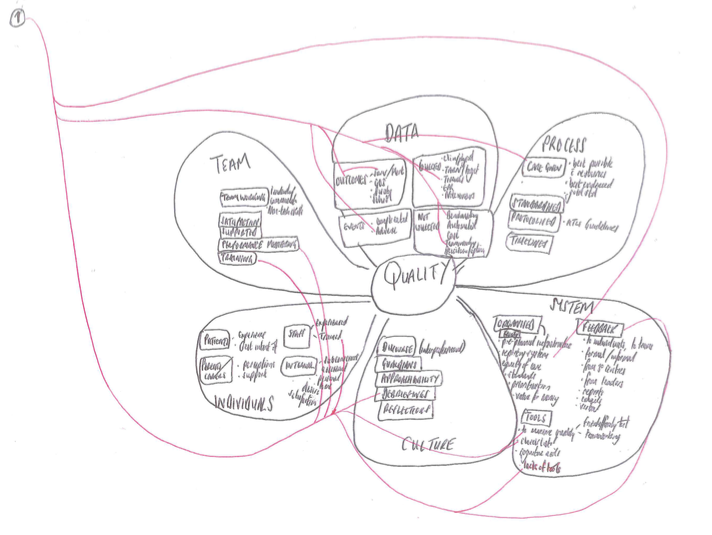

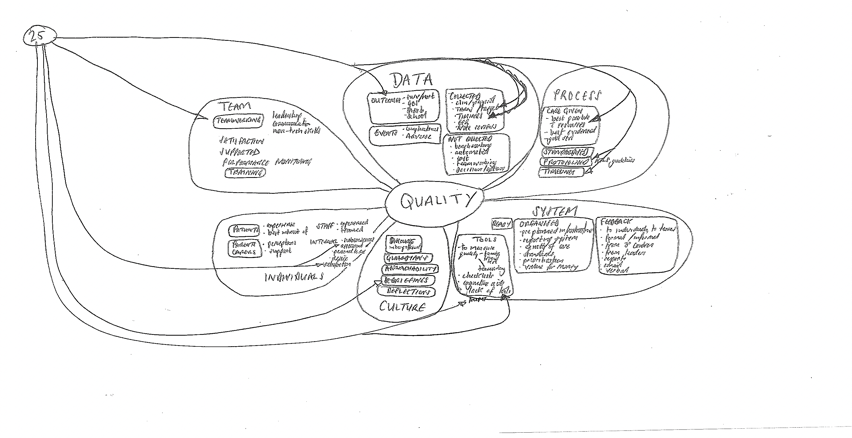


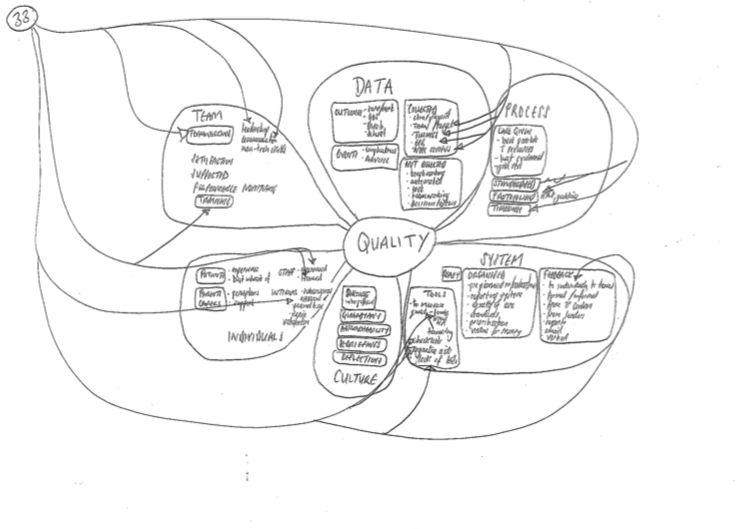


Exploration of the hierarchy of perspectives
This resulted in a hierarchy of perspectives from a simple to a complex level.


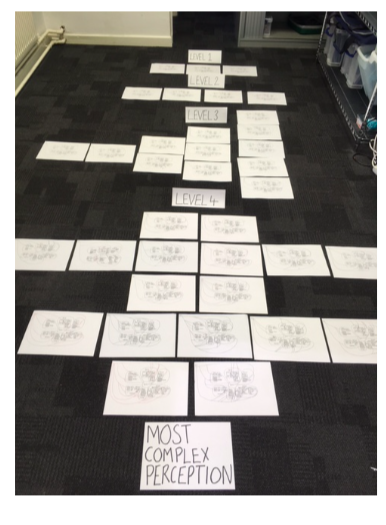


Numerical Analysis
This resulted in a table of the counted meaning units of quality per participants per role (trauma team or administrator).


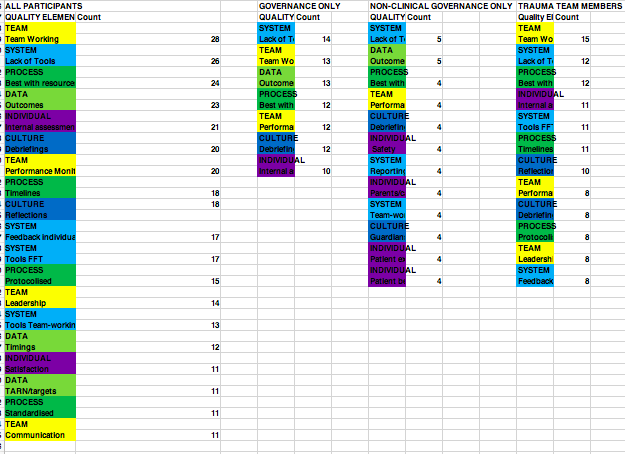

Supplement: Supplementary file 4 — The phenomenography data audit trail (DOCX 1553 kb) [file 41077_2019_91_MOESM4_ESM.docx]
