# Supplementary material for: Defining and measuring quality in acute paediatric trauma stabilisation: a phenomenographic study
Source: Adv Simul (Lond). 2019 Apr 11;4:4. doi: 10.1186/s41077-019-0091-z (PMC6458622; doi:10.1186/s41077-019-0091-z)
Supplement: Supplementary file 5 — Paediatric trauma educational interventions targeted to directly improve patient care—participant information sheet (DOCX 82 kb) [file 41077_2019_91_MOESM5_ESM.docx]

**Paediatric Trauma Educational Interventions Targeted to Directly Improve Patient Care – Participant Information Sheet**

Investigators:

Prof Ralph MacKinnon, Royal Manchester Children’s Hospital, Manchester, UK & Karolinska Institutet, Sweden.

Dr Chris Kennedy, The Children’s Mercy Hospital, Kansas, USA

Dr Terese Stenfors, Karolinska Institutet, Sweden

Purpose of the Research:

The overall goal of the project is to determine the key individual team member, team and hospital system factors and interactions during the initial stabilization of pediatric trauma patients that can be modulated and improved with in situ simulation training. This will lead to the development of an evidence based pediatric trauma simulation training toolkit to assess and recommend improvements in trauma team – hospital system-based interactions.

The ultimate objective is then to study the effectiveness of the training program on the direct patient care provided.

Description of the Research:

- You will have the study explained to you in person and given an opportunity to ask questions before consenting to take part
- You can withdraw your consent at any point
- Your anonymity will be maintained throughout this project & thereafter
- There will be no recording of individual name / personal data or institutional identifiers
- This study will involve you participating in a 60-minute interview
- You will be asked for your thoughts and suggestions regarding quality improvement by a global paediatric research and education collaboration.
- All interviews will be audio recorded for later analysis.
- The interview data will be transcribed and stored in a secure location. This data will only be seen by the principle investigator and by research colleagues who are from North America, New Zealand or Sweden.
- The data collected may be used for directly related research.
- At your request, you can receive a copy of the study results at the end of the study.
- Participation is entirely voluntary and decision to participate or not will not affect your working life in any way.
- The decision to participate or not will in not be shared with others.

Potential Harms:

We know of no harm that taking part in this could cause you. Neither your performance nor your non-participation will be used towards any work place evaluation in any way. You are free to withdraw from the study at any time. We will inform you of any new information that might influence your decision to continue to participate in this research project.

Potential Discomforts or Inconvenience:

The aim is to participate in the interview within your usual working time.

Potential Benefits:

Involvement in this study will be of no direct benefit to you, although the participation may be seen as a refresher of paediatric trauma quality improvement methodology.

The research results will be made available to you at the end of the study.

To society: If the FACT is found to be fit for purpose then this will allow evaluation of targeted in situ / point of care educational, decision support and training to be assessed in terms of directly improving trauma care then this has broad implications for the improvement of the management of traumatically injured children.

Alternatives to participation:

You are not obliged to participate in this study.

Confidentiality:

We will respect your privacy. No information about who you are will be given to anyone or be published without your permission, unless required by law.

The data produced from this study will be stored in a secure, locked location. Electronic data will be securely encrypted. Only members of the research team (and potentially research quality monitors) will have access to the data. North American, New Zealand, Swedish reviewers will observe and rate the UK video data. This could include external research team members. Following completion of the research study the data will be kept as long as required then destroyed as required by Royal Manchester Children’s Hospital policy. Published study results will not reveal your identity.

A copy of this consent form will be given to you as the participant.

Reimbursement:

There will be no out of pocket expenses for being in this study.

Participation:

It is your choice to take part in this study. If you choose to take part in this study, you can take yourself out of the study at any time.

Sponsorship:

The study is Sponsored by Central Manchester University Hospitals NHS Foundation Trust.

Conflict of Interest:

Professor MacKinnon and the other research team members have no conflict of interest to declare.

Who has reviewed the study?

The R&I Division within Central Manchester University Hospitals NHS Foundation Trust has reviewed this study.

Further Information

If you would like any further information or have any questions about the study, please contact:

Prof Ralph James MacKinnon, Dept. of Paediatric Anaesthesia, Royal Manchester Children’s Hospital, Manchester, M13 9WL, 0161-701-1263, [Ralph.mackinnon@cmft.nhs.uk](mailto:Ralph.mackinnon@cmft.nhs.uk)

If you would like any other general advice or information about taking part in research, please contact:

Alison Robinson, Division Research Manager, Royal Manchester Children’s Hospital, Hathersage Road, Manchester, M13 9WL, 0161-701-2933

If you have cause for complaint about the conduct of this study:

please contact:

Dr Lynne Webster, Head of Research Office, 1st Floor Postgraduate Centre, Manchester Royal Infimary 0161-276-4124, [lynne.webster@cmft.nhs.uk](mailto:lynne.webster@cmft.nhs.uk)
